# From Eminent Men to Excellent Universities: University Rankings as Calculative Devices

**DOI:** 10.1007/s11024-017-9329-x

**Published:** 2017-06-28

**Authors:** Björn Hammarfelt, Sarah de Rijcke, Paul Wouters

**Affiliations:** 1 Swedish School of Library and Information Science, University of Borås, Allégatan 1, 501 90 Borås, Sweden; 2 CWTS, Leiden University, 2333 AL Leiden, The Netherlands

**Keywords:** University rankings, Calculative devices, Excellence, Eugenics

## Abstract

Global university rankings have become increasingly important ‘calculative devices’ for assessing the ‘quality’ of higher education and research. Their ability to make characteristics of universities ‘calculable’ is here exemplified by the first proper university ranking ever, produced as early as 1910 by the American psychologist James McKeen Cattell. Our paper links the epistemological rationales behind the construction of this ranking to the sociopolitical context in which Cattell operated: an era in which psychology became institutionalized against the backdrop of the eugenics movement, and in which statistics of science became used to counter a perceived decline in ‘great men.’ Over time, however, the ‘eminent man,’ shaped foremost by heredity and upbringing, came to be replaced by the excellent university as the emblematic symbol of scientific and intellectual strength. We also show that Cattell’s ranking was generative of new forms of the social, traces of which can still be found today in the enactment of ‘excellence’ in global university rankings.

## Introduction

The American psychologist James McKeen Cattell (1860–1944) published the first university ranking in November 1910. His calculation of the ‘Scientific strength of institutions’ listed American universities in descending order depending on the number of eminent scientists employed. Some hundred years later university rankings have become increasingly important devices for assessing the ‘quality’ of higher education and research. Ever since the arrival of the first international university ranking in 2003, the *Academic Ranking of World Universities* developed by Shanghai Jiao Tong University, rankings are an integral part of discussions regarding the role and impact of universities.

The emergence of university rankings is often understood as resulting from a drive for more accountability and transparency in the governance of higher education. This demand-, or pull-driven explanation is common in current research on rankings, and the same line of reasoning is also found in earlier studies (Webster 1986). According to Hazelkorn (2011: 6–9), four main ‘drivers’ of university rankings can be identified: the positioning of knowledge as the foundation of economic, social and political power; increasing demographic pressure in many countries; higher education seen as a vital component for an industrious nation; and the emergence of the student as a ‘savvy consumer.’ These drivers might indeed be viable explanations for the popularity of university rankings and their presence in discussions on the issue of quality assessment in the governance of science and scholarship. However, as principal reasons for the development of rankings they put too little emphasis on the ranking practices and methods employed. Rather, when scrutinizing their history we find that university rankings, local as well as international, often were initiated for completely different reasons than transparency and accountability. Strategic considerations of the institutions using the ranking as well as the availability of more data about research and teaching performance have resulted in the perception of new rankings as reputational and management tools. But the emergence of rankings is also strongly intertwined with the advancement of research in fields such as statistics and bibliometrics. Consequently, we argue that the emergence of rankings can only partly be understood as an answer to pull-driven demands and developments—internationalization, globalization and economization—in higher education. Furthermore, the popularity of university rankings cannot solely be explained by increasing top-down governance in neoliberal academia, because the practice of ranking ties in with deeply engrained cultural repertoires around competition and performance. Arguably, ‘to rank’ comes natural for professions that have become highly competitive of themselves (de Rijcke et al. 2016: 8). The history of rankings, the epistemological logic behind them and their disciplinary background are crucial elements often overlooked in contemporary research. In this sense we agree with Marginson (2014: 47) that surprisingly little critique is directed towards rankings as social science, although the methods of ranking are taken from disciplines such as sociology, psychology, economics and information science. Thus, the current study supplies a much-needed ‘push’ perspective on a phenomenon usually explained by ‘pull’ mechanisms.

An explanation for the success of rankings originates from their ability to make heterogeneous characteristics of universities comparable through classification, normalization and standardization (de Rijcke et al. 2016), thus allowing universities to take part in a global higher education market driven by a competition for expertise, reputation, students and money. Here our main interest lies not so much in the conceptualization but in the concrete enactment of ‘comparability.’ We will show that rankings as a social technology make highly diverse entities—like universities—measurable through quantitative methods; or in other terms, rankings make universities ‘calculable.’ This practice or ability of making calculable is the main topic of our study. Calculation is defined as a process of making distinctions between actors (things, institutions, states), and to determine possible actions and consequences associated with these actors. As argued by Callon and Muniesa (2005), such a broad definition is useful in avoiding a sharp distinction between judgment and calculation. Drawing on Latour (1987) and his concept of ‘centres of calculation,’ the framework formulated by Callon and Muniesa also highlights the movement of materials, which allows for a detailed analysis of the material practices of ranking. In order to develop a better understanding of how universities are made calculable through rankings, we focus on the construction of ‘eminence’ in the socio-technical context in which the first university ranking emerged. In particular, we adopt the notion of ‘calculative devices’ (Callon and Muniesa 2005) to demonstrate the interaction between the ways in which scientific ‘eminence’ was defined and the means and methods to measure it. The process of making calculable is then exemplified by the first university ranking. In this part of our paper we link the epistemological rationales behind the construction of Cattell’s first ranking to the sociopolitical context in which he operated: an era in which the field of psychology became institutionalized against the backdrop of the eugenics movement and the rise of statistics as a legitimate means to assess ‘innate’ eminence. Secondly, the scientific and socio-political context in which the first ranking emerged enabled a redefinition of universities and scientists as ‘commodities’ on an international market for ‘eminent’ institutions and scholars. This market afforded space to an intricate valuation system for scientists and institutions on the basis of their respective status. Third, we demonstrate how this measurement practice not only reflects contemporary social values and scientific norms, but is also generative of particular forms of the social itself (cf. Moor and Lury 2011). The ways in which Cattell combined certain measurement practices with peer evaluation among ‘eminent’ men also created highly novel ways of valuing scientific institutions. These novel understandings of eminence arose in tandem with the new ways in which eminence was recognized and measured. In fact, the act of measuring eminence defined what would henceforth count as eminence. As a result, the concept lost the ambiguity it could still retain when it was not yet stabilized by the process of measurement. Comparable processes of definition and social construction can now be seen around contemporary enactments of excellence and quality.

In a concluding section, we relate the history of the first university ranking to contemporary developments by showing how the operationalization of ‘excellence’ is shaped by epistemological concerns, methodological choices and the availability of instruments.

## Background

Though global university rankings are a quite recent phenomenon, the literature on the topic is immense. Hazelkorn (2011) estimates that there are over one thousand papers and books on the theme of rankings and the number of publications has surely increased since the publication of her book. To even begin to review this vast literature is a daunting task, but a few strands in the literature can be outlined here. Generally we find three types of studies on the topic: critical studies, methodological studies and studies of influence and effects. These literatures rarely overlap. Critical studies usually focus on the ideological level, and rankings are discussed as an indication of a more general development described under headlines such as ‘marketization’ (Bok 2009), ‘commodification’ (Radder 2010), ‘academic capitalism’ (Slaughter and Rhoades 2004) or ‘neoliberalism’ (Shore 2010) (for a recent example, see Pusser and Marginson 2013). Usually, these studies have their origin in research focused on developments in higher education more generally, and they tend to focus on the phenomenon of university rankings rather than on methodology used or construction of rankings.

The second strand of research tests university rankings empirically. Here, the methodologies of university rankings are in focus, indicators used and calculations made are scrutinized. An early example is Van Raan (2005) who pointed to several problems in the ARWU-ranking: bias towards US journals, bias towards sciences well-covered in *Web of Science* and bias towards English language journals. In-depth methodological studies and criticisms pertaining to technical aspects have pointed to the many problems associated with calculating the quality of universities (Bougnol and Dulá 2015), and attempts of replicating rankings are common (e.g., Docampo 2012).

The third line of research focuses on the effects of university rankings, using concepts such as ‘performativity’ (Callon 1998) and ‘reactivity’ (Espeland and Sauder 2007, 2016) to describe how rankings influence behavior. These studies often build on interviews or questionnaires targeting various stakeholders (Hazelkorn 2011; Collins and Park 2016) or analysis of rankings in popular media (Wedlin 2006).

The history of university rankings is a less developed line of inquiry compared to the major directions outlined above. Cattell’s role as the creator of the first proper university ranking was noticed already by Webster (1985, 1986), but it was Godin (2006, 2007) who firmly connected these efforts to Francis Galton and the eugenics movements. This study latches on to these accounts of the development of university rankings, and we point to parts of the history that have been overlooked in earlier research, such as Cattell’s early use of doctorates to measure ‘scientific strength’ and how the ranking was reported in the press. Most importantly, however, we introduce the concept of ‘calculability’ in order to align the practical construction of the ranking with the historical context in which it was produced.

## Ranking and Calculability

Our conceptualization of university rankings as calculative devices, which contribute to the establishment of a market, fits well with a contemporary debate on commodification and academic capitalism. Ideas concerning a market for universities and academics might appear as more far-fetched in the context of the early 20th century, and the enactment of the first university ranking. Yet markets, both for students and scientists, were not only explicitly discussed, but were also an important reason for constructing a ranking in the first place. A narrow focus on ‘market devices’ might, however, restrict our analysis to the use, or intended use, of rankings. We show that rankings are also fundamentally grounded in contemporary practices of knowledge production. We think it is crucial to consider this particular register and the concomitant regimes of valuation in accounting for the omnipresence of rankings.

Callon and Muniesa (2005) explain calculability in three steps: First, the entities supposed to be calculated are detached from their original context. This can be understood through the process of singularization described as follows: The entity (good) is detached from its production, a process made possible through the objectification of the good, then it is adapted to the world of the buyer, which in turn is arranged to receive it. Finally, it is integrated in the social and technical networks of the buyer (Callon and Muniesa 2005: 11). The ‘citation’ is a fine example for showing how detachment takes place in the case of rankings: the reference—supplied by the producer/author—is turned into a citation through its inclusion in a citation index and becomes entangled in a ‘citation infrastructure’ where it eventually comes to represent scientific impact (Wouters 2014).

The reduction of more complex valuation practices into numerals is here an important part of the process, as numbers are both transferable and easily communicated to a large audience. Furthermore, quantification is one of the most effective strategies for turning social arrangements credible, objective and impeccable (Porter 1995). Their abstractness also reduces the need for specific, in-depth knowledge and enhances the universality of measurement. Furthermore, numbers are both easily decontextualized, and re-contextualized, which allows for them being used for new purposes in novel contexts (Espeland and Sauder 2007: 18).

In the second step, things are sorted out and related to each other. Thus, detached items, such as citations, publications, the number of Nobel prizes, external grants received and so forth are now compared and linked with each other. Callon and Muniesa (2005: 15) describe this as “[…] a process of classification, clustering and sorting that makes products both comparable and different.”

Finally, a result has to be produced and presented for a calculation to be completed. In this step the different indicators are integrated to form a descending list of entities. Deliberations and calculations used to reach this particular order are black-boxed, and presented as a uniform ranking of universities. Hence, the production of university rankings can be divided into three distinct practices using this theoretical framework: *detaching*, *sorting*, and *presenting*. This arrangement allows us to deconstruct the first proper university ranking and describe the necessary steps needed for its production. The artifacts, or the ‘calculative spaces’ (Callon and Muniesa 2005), through which rankings are produced is here of particular interest, and directories, paper slips and lists are examples of devices used by Cattell. Furthermore, our analysis underlines how ideological underpinnings, technical procedures, methodological considerations, epistemic criteria as well as dissemination channels came to shape the first university ranking. The theoretical structuring we adhere to also demonstrates how alternative approaches were gradually abandoned; for example, how the method of measuring scientific strength using the number of doctorates was discarded. At each of the three steps choices were made—on method, scope, and presentation—and the final published ranking was the product of these considerations. A decisive reason for adopting a theory of calculability is also its potential for analyzing rankings more generally: with a few modifications the structure and theoretical framing used in this study can also be applied to the construction of contemporary rankings.

## James McKeen Cattell, and His Work on *American Men of Science*

James McKeen Cattell (1860–1944) was an American psychologist and long-time editor of *Science*. Cattell was trained in Europe and held a lecturing position in Cambridge, where he came into contact with the eugenics movement and the famous statistician Francis Galton, before he became a professor at Columbia University in 1889 (Sokal 1980). The scientific methodology of the eugenics movement provided Cattell with the tools for imagining, classifying and acting on ‘eminence’ differently, by linking novel forms of measurement to new ways of valuing scientific performance (cf. Moor and Lury 2011). This eventually resulted in the invention of the first university ranking.

The main problem for Cattell, and many contemporaries interested in eminence and greatness, was a perceived decline in great men compared to earlier periods (Godin 2007). A fear of a biological degeneration of the population was an important motivation for the eugenics movement, as inheritance of mental abnormalities was believed to result in a larger proportion of the weak and insane. A general anxiety regarding the decline of the British Empire further intensified the interest in heredity (Waller 2001).^1^ The fate of the nation was dependent on the overall quality of ‘men’ and the measurement and promotion of eminence was deemed as an important task.

Late 18th-century interest in great individuals should also be viewed in the light of one of the major anxieties of the time; the fear of the masses. The advent of industrialism, democratization and growing working class were all contributing to this fear. Influential thinkers, such as the French physician Gustave Le Bon, claimed that human beings lost their ability to think independently in the crowd, as their individual selves were absorbed by a ‘crowd soul’ (Jonsson 2008)^2^. The eminent, freethinking individual thus became increasingly important as a protagonist of rational thinking and human advancement.

Furthermore, the preoccupation with great men was accompanied by a growing interest in the role of the scientist in a time when scientific work to an increasing degree became an occupation among others (Shapin 2008: 21–46; Baldwin 2015: 4–20).

In his early work Cattell supported the notion that greatness is inherited rather than acquired: “The little scientist can doubtless be made, but probably the great man of science must be born” (Cattell 1903c: 567). However, he quite rapidly changed his view on the nature-nurture debate, and just three years later Cattell interprets his own studies of scientists as proof against the hereditary view.^3^ Thus, the eugenics movement and the ideas associated with it were highly influential for Cattell and his work on the distribution of eminence, but gradually he began to question some of his initial beliefs (Godin 2007). Cattell’s change of position, from nature to nurture, also meant a shift in the focus of his study, where the institutions of science came to take center stage.

The work of Galton spurred Cattell to study differences between humans with regards to intellectual ability. He started with a brief study on exceptional ability, where he listed the thousand most eminent men, based on the space they occupied in dictionaries and encyclopedias (Cattell 1895). These investigations were later developed and published under the title “A Statistical Study of Eminent Men” in 1903. In this work, Cattell examined six biographical dictionaries and encyclopedias using the length of the bibliographical note as an indicator of eminence. He admits that the selection of men having “attracted the eyes and ears of the world” is a crude measure - the most ‘eminent’ man, Napoleon, was according to Cattell neither a “genius nor a great man” (Cattell 1903: 361)^4^. Instead of providing a definition of ‘eminence’ *a priori*, Cattell trusted that his objective and impartial method would guide him. Thus, the methodology of taking the length of the note as a form of value made sense for Cattell because he treated eminence and reputation as interchangeable - as Galton had done before him (Godin 2007).

With the first edition of *American Men of Science*: *A Biographical Directory* (1906a), Cattell once again followed in the footsteps of Francis Galton who had published his *English Men of Science* in 1874. According to Cattell, an important reason for publishing this reference work was to “[…] make *men* of science acquainted with one another and with one another’s work” (Cattell 1906a p. V [our italics]). Women were almost absent in the discourse on eminence; the directory instigated by Cattell changed its name to *American Men and Women of Science* as late as 1971.^5^ For Galton, the main contribution of women was as able mothers of eminent men. Cattell is clearly misogynist in his early work, where he finds a biological explanation for the lack of women among eminent scientists: “Women depart less from the normal than man—a fact that usually holds for the female throughout the animal series; in many closely related species only males can be readily distinguished” (Cattell 1903: 375). Also in later studies Cattell tends to explain the poor representation of women, 18 out of 1,000 scientists, as a result of innate differences between the sexes (Cattell 1910: 676). However, in his later writings Cattell pondered on the unrealized potential that women offered for science, especially where he suggests that “it is possible that the lack of encouragement and sympathy is greater than appears on the surface” and he considers the prospect of a future in which women’s contribution to science matches that of men (Cattell 1910: 676).

The four editions of *American Men of Science* that Cattell edited became his main material for studying science.^6^ The essay that accompanied the directory tells us a lot about the background, including data on birthplace, city, age, and most notably the college or university attended by researchers, and the methodology he used. The directory of eminent men also served other purposes. For example, the analysis of the distribution of scientific eminence across cities and universities was a primary goal: “…we can tell whether the average scientific standard in one part of the country, at a given university, etc., is higher or lower than elsewhere; we can give quantitatively, the men being weighted, the scientific strength of a university or a department” (Cattell 1903c: 567). According to Godin (2007), statistics were collected to address this problem, and to contribute to the progress of science. What Cattell was aiming for was nothing less than a “[…] natural history or ecology of men of science” (1903c: 562).

## Making Universities Calculable: The Construction of the First University Ranking

When studying the ‘ecology of scientific men,’ Cattell found that one particular institution - the university - played an important, if not crucial, role for the advancement of science. In this respect he clearly distanced himself from Galton, who had had little praise for universities and their role in fostering eminence in his *English Men of Science* (1874).^7^ Their departing view was partly due to differences in their positions in the nature or nurture debate, where Cattell eventually came to downplay the role of inherited giftedness. In this context, universities became a natural point of departure when studying the distribution of scientific men. Eventually, his interest in the institutions of science came to result in a ranking, where the overall scientific strength of American universities was presented.

As noted above, three procedures—detachment, sorting and presenting were needed before a calculation of institutional eminence could be performed. Although we find it useful to structure the analysis according to these three steps, it is important to stress the blurriness of these practices. Initial categorizations of researchers are presented already in the detachment stage as names are pre-sorted based on disciplinary affiliations, and all three procedures are present throughout. Moreover, the process is not in any way linear. Cattell presented his results continuously, and the assumptions underlining his project and the aim of his undertaking changed over time in an iterative process.

### Detachment

**Fig. 1:**
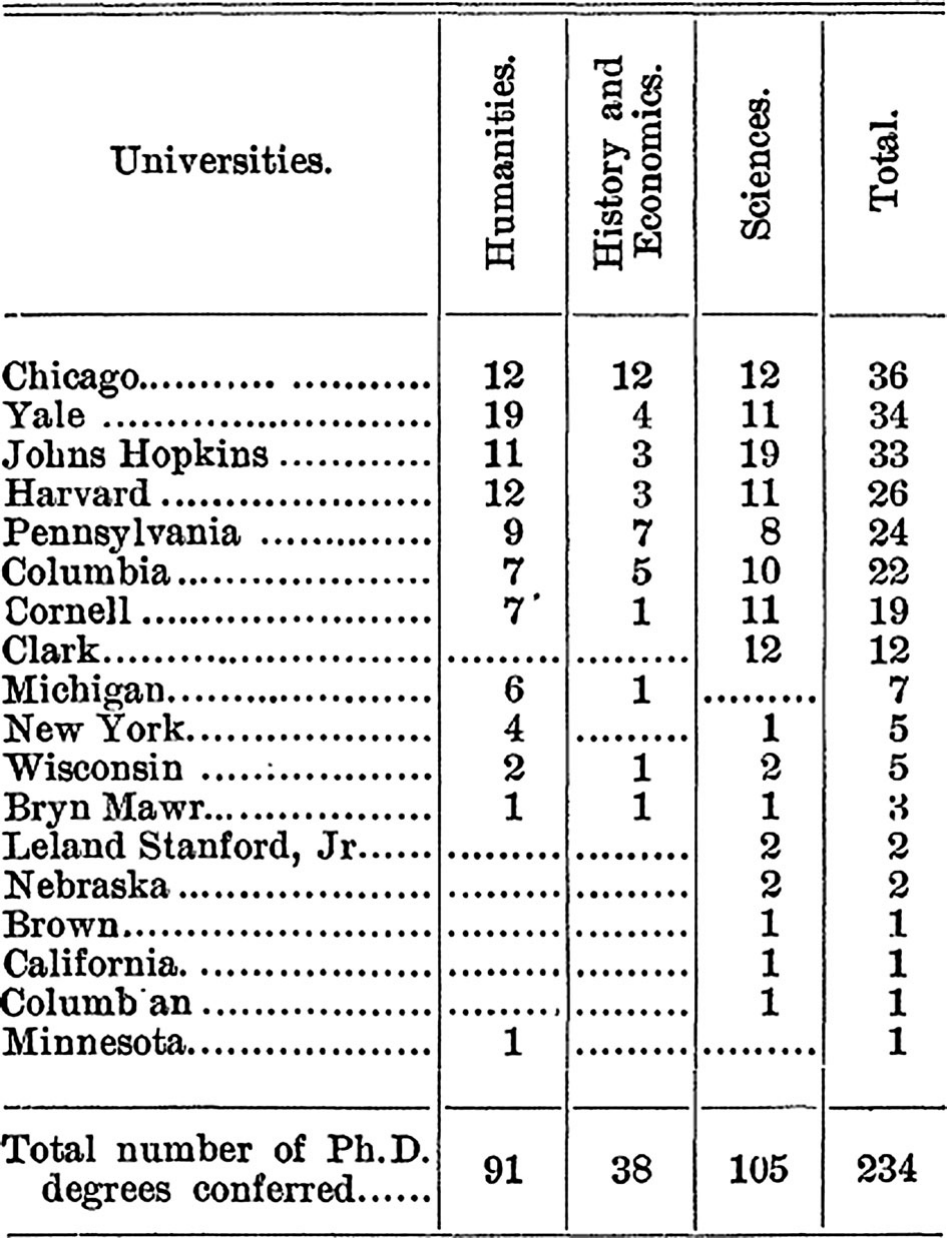
Order of universities by the number of doctorates awarded. From “Doctorates Conferred by American Universities for Scientific Research.” Reprinted with permission from AAAS (Cattell 1898: 198)

Reputation was Cattell’s preferred indicator in measuring scientific merit, and this becomes apparent in his attempts of ranking institutions. Still, this choice was not obvious and he considered several other indicators of eminence. Of note are the early attempts of measuring institutional performance based on output of doctorates. Cattell states that the number of doctorates can be seen as important output: “The American university is definitely a place for research, where both teachers and students are engaged in research or in learning the methods of research. The results of the work of the students is in large measures summarized by theses for the doctorate, and it is interesting to know what is the outcome of the past years research” (Cattell 1898:197). Thus, more than ten years before the publication of his 1910 ranking Cattell presented an ‘Order of universities’ based on doctorates. From 1898 onwards he published a list of the number of doctorates awarded in the US (Fig. 1). The current history of Cattell’s work and the construction of the first university ranking has mostly ignored these earlier attempts of measuring institutional performance based on output.

It could be argued that the list of universities building on the number of doctorates awarded in fact was the first ranking of universities. The list of doctorates conferred was published annually. Results from earlier years were reproduced and compared in subsequent editions and results were presented in descending order. Thus, it allowed for comparing the output of universities over time. In 1903, Cattell explicitly discusses the position of different institutions: “It will be noticed that five universities are distinctively in *advance*, and that a large majority of the degrees—four fifths are conferred by seven universities. There has been no considerable *change in the positions* of the universities during the years covered by records, though there is apparently an increase at Columbia and Michigan and a decrease at Johns Hopkins and Clarke” (Cattell 1903b: 258, our italics).

In his work on eminent men, Cattell realized that his data could be used to rank universities, and the same applies to the counting of doctorates. In the 1909 article on *Doctorates Conferred by American Universities* he not only explicitly refers to “the change of position of the leading universities” but he also uses the number of scientific men as an additional indicator to substantiate his findings (Cattell 1909: 227).

Cattell gradually understood that the number of graduates, or the reputation of individual scientists could be detached from their original context and be used as indicators for the ranking universities. He also envisioned that these indicators could be combined—although he never formally did so in a proper ranking. In the 1910 article introducing the first known ranking Cattell also discusses the possibility of using the number of doctorates as a proxy for ‘scientific strength’: “We may perhaps assume that the relative strength of a university in different departments tends to be proportional to the number of research degrees conferred” (Cattell 1910: 684). The cautious words, “may perhaps,” used by Cattell are one possible explanation as to why he never used doctorates to construct a proper ranking. It might be that he saw mere counting of heads, or number of published papers, as crude indicators of ‘scientific strength.’ In fact, he published several lists of the number of scientists, doctorates and scientific papers produced at American universities but these were never presented as measures of overall strength. It was not until he was able to add and calculate the qualitative factor of peer judgments that he proceeded to construct a ranking. Hence, although Cattell in many instances hints at doctorates or papers being an indicator of ‘strength,’ he never advertised these lists as actual rankings. The same applies to earlier lists of universities based on the eminent men attending them, which were produced by scholars working in the eugenicist tradition (cf. Maclean 1900; Ellis 1904). Making lists involves a process of decontextualization. They enforce boundaries and create hierarchies (Goody 1977), and making a list thus marks an important step in the construction of a ranking. A ranking is, however, something more than a list. As pointed out by Webster (1986), a university ranking should be arranged based on a specific criterion that the compiler believes to reflect the academic quality of the institution in question. Thus, the doctorate lists come close to being a ranking in a formal technical sense but they were not constructed nor presented as such.

Instead, Cattell came to construct his ranking based on his study of *American Men of Science*. Crucial for this undertaking was the qualitative judgment made on the relative standing of scientists made by peers. Building on his initial list of prominent scientists—derived from university rosters, scientific journals and bibliographical dictionaries—Cattell asked ten prominent representatives from 12 disciplines to rank individuals according to merit. These representatives were then presented with an initial list of men “known to have carried on research work of any consequence” (Cattell 1906d: 660). According to Cattell, the number of pre-selected names was proportional to the total amount of researchers within a given field: ranging from 175 in chemistry to 20 in anthropology. The representatives were provided with slips containing names and addresses of scientific men being ranked. The instructions for ranking describe the procedure in detail:

In case there is noted the omission of any scientific man from the list who should probably have a place in the first three quarters, a slip may be added in the proper place with his name and address. In case there are names on the list regarding which nothing is known, the slips should be placed together at the end. The slips, as arranged in order, should be tied together and returned to the undersigned. (Cattell 1906b: 661)

The list as a technique for ordering information was of great importance for this maneuver, as it allows judgments on the quality of individual researchers to be stabilized and visualized. This operation is a pre-requisite for calculation as it transforms mainly oral statements into visual ones (Goody 1977: 106). In this case stabilization is enacted through slips that are ‘arranged’ and ‘tied together’ to form a ranked list of notable scientists in each field.

Cattell understood that this operation was not straightforward and his instructions point to two complications that also remain troublesome in contemporary rankings. The first concerns how to deal with interdisciplinarity: “[…] an eminent astronomer might also be a mathematician, but in ranking him as a mathematician only his contributions to mathematics should be considered” (Cattell 1906b: 661). Making disciplines comparable was another difficulty encountered when arranging the order of scientists. Cattell notes that a scientist falling between the disciplines is “[…] likely to receive a lower position than he deserves” (Cattell 1906b: 664).^8^ He also discusses the problem of self-evaluation (e.g., ranking yourself) and found that scientists were equally inclined to overrate as to underestimate their own contribution. Moreover, respondents were likely to overestimate the importance of close colleagues and to give higher ratings to researchers working on topics closely related to their own field of inquiry (Cattell 1906b: 664).

The thousand leading men of science ranked according to this method would be the basis for his statistical studies. Cattell continued with further categorizing the most eminent scientists into groups with a hundred men in each. When comparing the difference between the groups Cattell found that the distribution of merit was highly skewed where the first hundred had a scientific merit equal to the second and third hundreds together (Cattell 1906c: 707).

Cattell then proposed that his ranking of men could be converted into a ranking of institutions (Cattell 1910). The points given to individual scholars were aggregated at the level of institutions to produce a number indicating ‘scientific standing.’ Hence, the relative ranking of individual researchers was detached from its original use (to rank researchers within a given research field) and was employed for the purpose of ranking institutions. Yet, this maneuver was not a straightforward operation as numbers had to be sorted before the actual ranking could be produced. The sorting into cohorts based on overall position made researchers both different and comparable, which in turn allowed Cattell to develop a scientifically based ranking.

### Sorting

Building on his findings regarding the relative distribution of researchers, Cattell decided to award universities points depending on the position of their scientists among the top thousand. The development of a weighting system with points awarded according to the position of the scientists employed allowed him to produce a ranking of universities (Table 1).

**Table 1:** Cattell’s method for weighing the scientific strength of each university (Cattell 1910: 683)

Position of scientist	Points awarded
1–25	4
26–50	3
51–100	2.5
101–200	2.1
201–300	1.9
301–400	1.6
401–500	1.4
501–600	1.2
601–700	1.2
701–1000	1.0

This weighting was developed from the overall, and highly skewed, distribution of eminence that Cattell found in his studies. Hence, there was a scientific argument for assigning more points to highly ranked scientists. Support for this arrangement is given already when Cattell presented his 1906 study of eminent scientists. Summarizing his findings Cattell writes: “The first hundred men of science cover a range of merit about equal to that of the second and third hundreds together, and this again is very nearly equal to the range covered by the remaining seven hundred” (Cattell 1906c: 707). However, the decision to award researchers higher up the list significantly more points than those positioned further down was also based on actual salaries within universities. In fact, Cattell (1910: 683) asserted that salaries, on average, “increases with distinction and roughly measures it.” The pay structure within universities—where distinguished professors generally earned three times as much as assistant professors—provided further arguments for this arrangement (Godin 2007). Thus, both previous findings on the distribution of merit and the current pay structure within universities came to influence the methodology used for ranking.

### Presenting

**Fig. 2:**
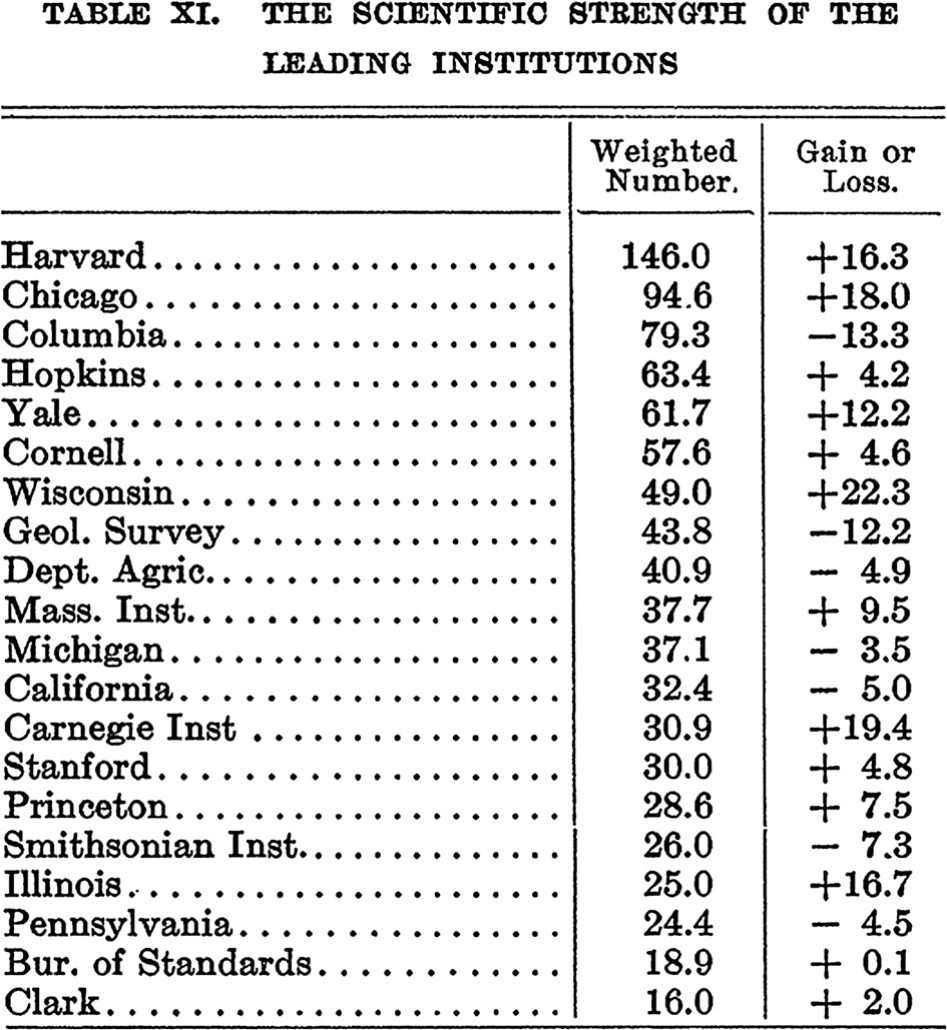
The scientific strength of leading institutions from “A Further Statistical Study of American Men of Science II.” Reprinted with permission from AAAS (Cattell 1910: 683)

On 11 November 1910 Cattell presented what he saw as the actual first ranked list of universities in *Science*. For the first time not only the number of eminent men but the quality (as judged by peers) of these men was presented in a ranked list of universities. Cattell argued that these numbers told us more than simple counting of men: “[t]hey take account not only of the number of men gained or lost, but also of the rank of these men and of the changes which have taken place through men improving their standing or failing to maintain it” (Cattell 1910: 683). Thus, the ranking did not only present a weighted number of scientific strength but it also indicated if a university was losing or gaining in the ranking (Fig. 2).

Cattell claimed that his ranking provided a fair estimate of the relative standing of the institutions on his list, and envisioned several possible uses. He believed that institutional rankings could inform students in their choice of university. This view resonates well with the present discourse on the topic. Another of Cattell’s concerns was international competition, which obviously also relates to current discussions. In Cattell’s case he discusses the standing of US science in comparison to Europe. However, his aim was not primarily that of national prosperity. Rather, he suggested that the US should contribute more to the global advancement of science (Cattell 1906d: 742). Thus, Cattell’s urge for international comparison was not primarily based on an assumption that American science should be better for the sake of the country, but rather that it should promote science as such.

Cattell’s third argument for using rankings is less visible in today’s debate. He suggested that university rankings could be used for raising the status and the salary of scientists. Scientists were underpaid compared to other qualified professions, and this was in Cattell’s view a major hindrance to science as otherwise talented men would choose other careers. One strategy of achieving this was through the production of rankings, of both men and universities. Thus, he started to calculate price tags for ‘scientific strength’: “A university can obtain a man of the first rank for from $5,000 to $7,500, or a man in the lower hundreds of the list for from $2,000 to $2,500” (Cattell 1910: 683). Clearly, Cattell imagined a market for academics where universities could invest in highly ranked scientists in order to boost their ‘strength’ and position in the ranking. He also suggests that competition for highly ranked scientists could be an important factor for the promotion of local universities (Cattell 1910: 688).

First of all, rankings could thus be used to empower researchers in the university system, which, according to Cattell, was increasingly run by administrators. Thus, his aims were linked to political purposes: emancipating university professors, and elevating their status in university administration. As such, there is an obvious link with Cattell’s fierce campaign, played out in ‘University Control’ (1913), and many pieces in *Science*, on problems of university administration and in particular what he perceived as a lack of authority and the right of say of university professors. This is fascinatingly similar to today’s debates where universities increasingly are seen to be managed by administrators (cf. Ginsberg 2011), and where rankings are presented as tools that could serve scientists and students. Cattell’s critique of the politics of his time targeted the manner in which universities were run.^9^ He particularly focused on the low status of the scientist in terms of freedom and in terms of monetary awards: “It seems to me that scientific men suffer in character because they are employees, rather than free men” (Cattell 1903c: 569–570). The tension between viewing the ideal scientist as an independent, curiosity-driven free individual, and advocating science as a respected and well paid occupation among others were ever present in Cattell’s thinking, and it related to a larger question of the time: Can independent and eminent men be grown in a dependent situation? Eventually, Cattell aimed for a compromise in which scientists were indeed employed, but with a high degree of autonomy; his ideal was the German research university of the 19th century (Godin 2007). Secondly, Cattell introduced ranking as a means to calculate and classify ‘eminence.’ This novel classification and calculation enabled a differentiation between distinct classes of merit, resulting in fundamentally novel forms of being ‘great’ with their own distinctive forms of ‘value’ attached to them (in terms of the salaries that should come with the different levels of ‘scientific strength’). Moreover, the classes of merit, and the salaries that should be based on them, were grounded in Cattell’s scientific studies of the distribution of scholarly merit. Similar to earlier studies of the distribution of eminence (cf. Galton 1869), Cattell found this distribution to be highly skewed.^10^

## The Legacy of the First University Ranking

The journal *Science* was Cattell’s main channel for advertising his study of American scientists, including his ranking. His biographical work, *American Men of Science,* was continually reproduced, and he also frequently published shorter notices on the number of doctorates awarded at major US institutions. However, Cattell did not reproduce his table comparing the ‘scientific strength’ of universities. A probable explanation for this is the lack of data. Though the biographical directory continued to be published, the method of letting peers rank researchers in their own field employed in 1903 and 1909 was not used in later editions. The sheer number of entries, growing from 4,000 in 1905 to 10,000 in 1921, also “greatly enhanced the labor and cost” of producing the directory (Cattell 1921: 118). As Callon and Muniesa (2005: 1233) also note, detaching objects, grouping, classifying and presenting them are actions demanding considerable effort and ‘calculative power.’ It may have been too much for Cattell. He did continue to follow the distribution of scientific men across universities and in an article introducing the 1927 edition he discusses changes in the distribution of scientific men (Cattell 1927: 513–516). Yet, due to lack of data only the total the number of esteemed scholars is counted and no calculation of overall scientific strength was presented.

The 1933 edition, the last one edited by Cattell, included a list of institutions where three or more of the starred scientists were employed (Cattell 1933: 269). Again Harvard is on top, followed by California and Chicago, but the list is not said to be representative for the overall quality of institutions and no ranked list of ‘scientific strength’ was presented. The tradition of comparing universities based on *American Men of Science* was later taken up by Stephen S. Visher who published a short piece in *Science* where the numbers for the institutions employing most of the leading scientific men are given (Visher 1945). He did not produce a descending list, but noted that Harvard ranks highest on the list of universities when it comes to “young starred scientists.”

The attention received by Cattell’s ranking is hard to estimate, and it may not have drawn the same relative amount of media coverage as today’s global rankings. However, Cattell’s study was reported by news outlets, and the implications of his study were discussed in a major piece in the *The New York Times Sunday Magazine* (20 November 1910). The article, “Americas Great Scientists rapidly decreasing,” was overall very supportive of Cattell’s efforts, calling his study “one of the most interesting documents the educational world has seen for many a day.”

An article published in the *Evening Star* titled “On the down grade” also focuses on the general decline of great men a cultural motif of the time: “[…] the present generation is decidedly below the mark set by its forerunners. The same that is true in literature, he says, will be found true in science if the test is applied throughout the world” (Cattell quoted in *Evening Star* Nov 24 1910). Thus, the general theme of a declining civilization which cannot match the greatness of previous periods is repeated.

Cattell was not the only one that took an interest in comparing universities at the beginning of the 20th century. In 1910, Edwin E. Slosson published *Great American Universities* in which 14 leading universities were compared on several criteria (including data on eminent men gathered by Cattell). Although Slosson did not present a uniform ranking of universities, his endeavor came close to current multidimensional rankings. 15 years later, Raymond Hughes was accredited with being the first to rank graduate programs in 1925 (Espeland and Sauder 2016: 9). These early attempts had in common that they originated from the sciences themselves and, although they claimed to be of relevance for students, their audience mainly consisted of fellow scholars.

What makes Cattell’s ranking stand-out compared to previous attempts of measuring scientific quality and to the rankings that followed? It was not only that he was first in presenting a hierarchical list that was explicitly said to reflect overall scientific strength. Cattell also pioneered two major approaches for quantifying academic quality; output-measures and reputational surveys. Furthermore, he utilized three main indicators used in university rankings today: manpower (number of doctorates), reputation among peers, and number of papers. Cattell also aligned measures of manpower with reputational measures in order to corroborate his findings. Moreover, the construction of the ranking grew out of a much larger scientific effort to understand the ‘nature’ of scientific eminence. Ranking for Cattell was not only a way of presenting his results, but it was also a method used for studying and influencing the scientific community.

## Discussion

Cattell’s notion of eminence, derived from the eugenicist tradition, influenced his choice of methodology, and was like contemporary rankings shaped by current conceptualizations of ‘excellence’ and ‘research quality.’ Cattell’s ranking built on the same basic idea about distribution as Galton in his *Hereditary Genius* (1869). Only a small sub-group (a thousand in total) of all scientists covered in *American Men of Science* were really considered to be outstanding, and of these an even smaller sub-group (the top hundred) were truly eminent.

Cattell arrived at two main and to some extent contradictory conclusions on the basis of these findings. First, he argued that his study could be used to discover factors and environments that are likely to foster eminence with the overall goal of increasing the proportion of ‘great’ scientists. In making this argument he clearly sees nurture (environment) as a more important factor than nature (inborn qualities). Yet, some of the proposed solutions—i.e., better salaries for married professors to encourage them to get more children and stipends for the children (sons) of professors - are in line with the idea of scientific eminence largely being innate.

Second, he argued for better conditions for these eminent men; they should be better paid, have more autonomy and better working conditions. The notion of a chain of institutions—from a world university where the truly outstanding scientists should be employed, a countrywide university for the nationally distinguished ones, and a local one for the less talented—is also part of the idea that the distribution of eminence among scientists should be reflected in institutional arrangements. These visions correspond not only to Galton’s thoughts on the distribution of geniuses, where only a selected few are truly great, but it also capitalizes on the idea of a meritocracy where every scientist is given a fitting position based on abilities and performance. In many ways this line of reasoning echoes the rhetoric of excellence that surrounds current day discussions on universities and their role in society. The ranking that Cattell presented effectively plotted the units of assessment on a scale with variable positions. This had important performative effects. Though the ranking clearly builds on the eugenicist idea that only a few people (and institutions) can be eminent, universities could from this point onwards hypothetically work themselves into a better position on the ranking. In effect, this new topography of eminence had a built-in potential to ‘nurture’ eminence. At the time, Cattell and contemporaries came to argue that the social [nurture] was more important than was first assumed. In the right environment, eminence was within reach even for those that were not born into it. This built-in (yet limited) potential to improve, we argue, is an important intellectual linkage between these ranking practices and the social science of the time.

The ideas that motivated Cattell’s ranking are crucial for understanding why and how the ranking was constructed. However, the role of instrumentation—lists, directories, surveys and slips—used to produce the first university ranking should not be underestimated. Similarly, citation databases, web-surveys and algorithms used in contemporary rankings play a significant role in the construction of ‘excellence.’ The directory *American Men of Science* was not developed to measure institutional strength, nor was the *Science Citation Index* designed for ranking journals or institutions. Rather, they were instruments designed to facilitate communication in science. Quite soon, however, the information these instruments provided was used for comparison and ranking. Hence, the availability of data determined to a considerable degree how rankings were constructed. Cattell pondered over the possibility of using the number of papers as an indicator of scientific strength. He considered, and made an attempt to produce, an international ranking of countries and institutions (Cattell 1926). He also had plans to reproduce his 1910 ranking, but the lack of adequate instruments for these purposes hindered him.

The rationale of the first university ranking was based on theoretical and methodological considerations from a long tradition of measuring eminence in the eugenicist tradition. The emergence of universities as the central hub of research as well as Cattell’s involvement in science policy and university politics came to redirect his focus from individuals to institutions. The ‘eminent man,’ shaped foremost by heredity and upbringing, came to be replaced by the excellent university as the emblematic symbol of scientific and intellectual strength.

The image of the researcher was highly unstable in the early 20th century, as older notions of the gentleman researcher driven by intrinsic motivations were juxtaposed with science as a paid profession. Cattell’s study of the ecology of scientific men, and his subsequent ranking of universities, was deeply rooted in this larger debate about the role of the scientist. Similarly, the current pre-occupation with university rankings can be related to a discussion regarding the multiple roles and instable identities of contemporary universities (Fallis 2007; Collini 2012). As we have seen, an important characteristic of rankings is the ability to define, make comparable, and stabilize heterogeneous objects through processes of detachment, sorting and presenting. Furthermore, the concept of calculative devices also questions a common separation of judgment and measurement as two distinct processes. As the example of the first university ranking shows, (peer) judgment is often a prerequisite for measurement. These elements are often overlooked in discussions that focus mainly on methodological issues, or on the role of rankings in relation to increasing competition in the academy.

We show in this study that deliberations at each stage were dependent on how excellence and scientific strengths were conceptualized when Cattell developed his ranking. At the detachment stage, for instance, the crucial question was which entity would have to be de-attached to come to represent eminence. Cattell deliberated between number of papers or number of doctorates, but eventually decided that reputation among peers was the best indicator for eminence. The peer evaluation was then detached from its original use of studying the distribution of eminence among individual scientists and employed for the purpose of measuring the scientific strength of institutions. In current rankings a range of proxies (citations, prizes, reputation among peers, student satisfaction etc.) can be singled out as representing qualities of a university, and each of these is based on specific perceptions of what these proxies represent. Then, the next question arises: How should these numbers be sorted so that they come to represent the combined quality of a university? As we have seen, Cattell developed a calculation procedure for this purpose based on the overall distribution of scientific men. Today, algorithms are used to weigh and normalize outputs (papers, citations, professors) in similar fashion. Finally, the results are presented, either as a definite descending list of institutions as in Cattell’s case, or, in a more multidimensional and less fixed manner (see, for example, U-multirank or the Leiden Ranking). Thus, notions of what constituted scientific strength underpinned each of the decisions made by Cattell, in ways very similar to how contemporary conceptualizations of ‘quality’ and ‘excellence’ are informed by current social values and scientific norms.

The idea that men, and universities, could be positioned on a single ranked list is apparent in the method used by Cattell. Scientists were asked to produce a single list, from the most prominent scientist downwards, based on a selection of names provided beforehand. The methodology chosen by Cattell latched on to existing ways of measuring eminence, developed in the eugenicist tradition of Galton and his disciples. However, the basic statistics available from *American Men of Science* was not enough for judging the strength of institutions. The mere production of scientists or papers could not be an indicator of eminence. Rather he saw reputation among peers as signaling true eminence. Hence, a detailed ranking of scientific men based on observations of the distribution of scientific merit among scientists became the preferred approach, as a ranking neglecting the highly skewed distribution of eminence could not be just. Consequently, his ranking was in many respects a by-product of his larger studies into the distribution of scientific men. Thus, the first ranking of universities in 1910 originated from an academic setting where the approach taken and methods chosen were supported by current research findings. Similarly, the first global university ranking was produced by researchers at Shanghai Jiao Tong University for the purpose of studying and improving the relative position of Chinese universities (Liu et al. 2005). The reputation and influence of commercial rankings largely rests on the use of scientific methods (for example, surveys and bibliometric analysis). Thus, both the first national and the first ‘global’ university ranking were not primarily produced for outsiders, but for academics themselves. Their development was not mainly driven by demands for external accountability and transparency that is advocated today, but by a more fundamental curiosity regarding the growth and progress of science.

## Footnotes

1 Similar fin-de siècle angst regarding the fate of Western civilization also manifested itself in continental Europe with Oswald Spengler’s *Der Untergang des Abendlandes* (1918–1922) as a prime example.

2 Le Bon’s most famous work, which laid the ground for the field of ‘masspsychology’ is *Psychologie des foules* (1895, or *The Crowd: A Study of the Popular Mind*).

3 “The inequality in the production of scientific men in different parts of the country seems to be a forcible argument against the view of Dr. Galton and Professor Pearson that scientific performance is almost exclusively due to heredity” (Cattell 1906: 734).

4 The ten most eminent men according to Cattell (1903a): Napoleon, Shakespeare, Mohammed, Voltaire, Bacon, Aristotle, Goethe, Caesar, Luther and Plato.

5 Cattell explains his use of the term ‘eminent men’ by a lack of more neutral concepts: “I have spoken throughout of eminent men as we lack in English words including both men and women, but as a matter of fact women do not have an important place on the list” (Cattell 1903a: 375).

6 Cattell would continue to be the editor of five subsequent editions of *American Men of Science* (1910, 1921, 1933 and 1938). The reference work has with the publication of the 2014 version reached 38 editions, and it changed its name to *American Men and Women of Science* in 1971.

7 “Much teaching fills a youth with knowledge, but tends prematurely to satiate his appetite for more” (Galton 1874: 257).

8 “It is more difficult to arrange the order when the work cannot readily be compared, as, for example, systematic zoology and morphology, but, as already stated, it is only expected that the arrangements shall be approximate” (Cattell 1906b: 661).

9 The political use of his study and the subsequent ranking is articulated already in 1910 when he ponders upon the relative losses and gains of different institutions: “It is, however, a fact not without significance that Columbia and California, in which faculty control is regarded by the administration as less important than executive efficiency, have suffered the most serious losses, whereas Harvard and Yale, where the methods of appointment and promotion are more democratic, show most gratifying advances” (Cattell 1910: 680).

10 Galton calculated that there were 400 Geniouses throughout history, one illustrious man per one million men and 250 eminent men per million (Godin 2007: 694).
